# Overexpression of *Magnaporthe Oryzae* Systemic Defense Trigger 1 (MoSDT1) Confers Improved Rice Blast Resistance in Rice

**DOI:** 10.3390/ijms20194762

**Published:** 2019-09-25

**Authors:** Changmi Wang, Chunqin Li, Guihua Duan, Yunfeng Wang, Yaling Zhang, Jing Yang

**Affiliations:** 1 State Key Laboratory for Conservation and Utilization of Bio-Resources in Yunnan, Yunnan Agricultural University, Kunming 650201, China; changmmiwang99@gmail.com (C.W.); chunq88@gmail.com (C.L.); dghpikachu@gmail.com (G.D.); wyunfeng1@gmail.com (Y.W.); 2001035@ynau.edu.cn (Y.Z.); 2 Key Laboratory of Agro-Biodiversity and Pest Management of Ministry of Education, Yunnan Agricultural University, Kunming 650201, China

**Keywords:** rice, *Magnaporthe oryzae*, effector protein, transcription factor, metabolites

## Abstract

The effector proteins secreted by a pathogen not only promote virulence and infection of the pathogen, but also trigger plant defense response. Therefore, these proteins could be used as important genetic resources for transgenic improvement of plant disease resistance. *Magnaporthe oryzae* systemic defense trigger 1 (MoSDT1) is an effector protein. In this study, we compared the agronomic traits and blast disease resistance between wild type (WT) and MoSDT1 overexpressing lines in rice. Under control conditions, MoSDT1 transgenic lines increased the number of tillers without affecting kernel morphology. In addition, MoSDT1 transgenic lines conferred improved blast resistance, with significant effects on the activation of callose deposition, reactive oxygen species (ROS) accumulation and cell death. On the one hand, overexpression of MoSDT1 could delay biotrophy–necrotrophy switch through regulating the expression of *biotrophy-associated secreted protein 4* (*BAS4*) and *Magnaporthe oryzaecell death inducing protein 1 (MoCDIP1*), and activate plant defense response by regulating the expression of *Bsr-d1*, *MYBS1*, *WRKY45*, *peroxidase* (*POD*), *heat shock protein 90* (*HSP90*), *allenoxide synthase 2* (*AOS2*), *phenylalanine ammonia lyase* (*PAL*), *pathogenesis-related protein 1a* (*PR1a*) in rice. On the other hand, overexpression of MoSDT1 could increase the accumulation of some defense-related primary metabolites such as two aromatic amino acids (L-tyrosine and L-tryptohan), 1-aminocyclopropane carboxylic acid, which could be converted to ethylene, vanillic acid and L-saccharopine. Taken together, overexpression of MoSDT1 confers improved rice blast resistance in rice, through modulation of callose deposition, ROS accumulation, the expression of defense-related genes, and the accumulation of some primary metabolites.

## 1. Introduction

In the natural environment, plants will encounter various pathogens, such as fungi, bacteria and viruses, which secrete effector proteins into plant cells to promote the colonization of pathogens [1,2,3,4]. During plant–pathogen interaction, plants can activate early defense responses such as callose deposition, early reactive oxygen species (ROS) accumulation and the expression of defense-related genes by recognizing effector proteins [5,6,7,8]. In addition, in order to adapt to biotrophy–necrotrophy switch (BNS) of hemibiotrophic fungi, plants will initiate multiple defense strategies, including the activation of ROS-scavenging related genes, the accumulation of defense-related metabolites, and the activation of jasmonic acid (JA) and ethylene (ET) signaling pathways [8,9,10].

Plant disease control in agricultural production mainly includes breeding disease-resistant varieties, chemical control and so on. Among them, identifying new disease-resistant resources and selecting new varieties with broad-spectrum resistance are the most economical and effective methods for disease control [11]. However, disease resistant varieties against hemibiotrophic pathogenic fungi remain limited [12]. Modern biotechnology, represented by genetically modified organisms, provides new strategies for the prevention and control of these diseases [4,13,14,15,16]. In recent years, it has been found that some transgenic plants could improve the broad-spectrum resistance of host plants; however, they may have negative effect on plant growth and development [14,17,18]. Overexpression of *Phytophthora sojae* crinkling and necrosis (CRN) effector (PsCRN115) [4], *Colletotrichum truncatum* effector (CtNUDIX) [7], *Magnaporthe oryzae* effector (MoSM1) [17] and so on could significantly improve plant disease resistance. In addition, overexpression of *Trichoderma viride* effectors (SM1 and SM2) and *Botrytis cinerea* effector (BcSpl1) could activate plant defense response and improve disease resistance in various plant species such as rice, cotton, maize, tobacco through regulating stomatal closure and salicylic acid (SA) signaling [14,19]. Different effector proteins secreted by *Magnaporthe oryzae* during rice–pathogen interaction can control plant physiological metabolism and inhibit plant defense response [20]. For example, biotrophy-associated secreted protein 4 (BAS4) plays a crucial role in the breakage of extra-invasive hyphal membrane of the host cells and biotrophic invasion of *Magnaporthe oryzae* [21,22]. *Magnaporthe oryzae* cell death inducing protein 5 (MoCDIP5) is specifically expressed in necrotrophic phase and induces host cell death [6]. Therefore, *BAS* and *CDIP* families can be used as marker genes of hemibiotrophic fungi to determine BNS [7].

In recent years, metabolomics has been widely used in investigating the metabolic responses in plant stress responses and defense responses [23]. The primary metabolic changes including amino acids, organic acid and sugars have been revealed in host plants against *Bipolaris oryzae*, *Magnaporthe oryzae* and *Nilaparvata lugens* [23]. L-tyrosine and L-tryptophan are called aromatic amino acids, which are related to the synthesis of multiple defense-related secondary metabolites [24]. Therefore, identification of defense-related metabolites such as aromatic amino acids can provide novel insight into plant defense response.

Recently, we identified an effector protein, *Magnaporthe oryzae* systemic defense trigger 1 (MoSDT1), which encodes C_2_H_2_ transcription factor, and obtained three independent transgenic lines overexpressing MoSDT1 in wild type (WT) variety of Ilimi [25]. However, the in vivo role of MoSDT1 in rice remains elusive, especially in plant defense resistance. To address this, the agronomic traits (the number of tillers and kernel morphology) and blast disease resistance between wild type (WT) and MoSDT1 overexpressing lines in rice were compared in this study. In addition, the effect of MoSDT1 overexpressing lines on callose deposition, ROS accumulation, defense-related genes and primary metabolites were also investigated, so as to reveal the underlying mechanism of MoSDT1 in plant disease resistance.

## 2. Results

### 2.1. MoSDT1 is a C_2_H_2_ Transcription Factor

**Figure 1:**
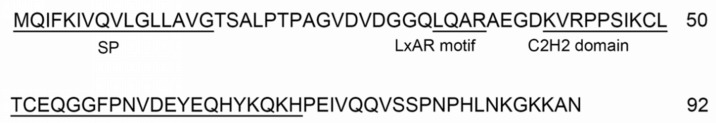
The conversed domain of *Magnaporthe oryzae* systemic defense trigger 1 (MoSDT1). Signal peptide (SP), LxAR motif and C_2_H_2_ domain are underlined.

In the previous study [25], MoSDT1 was firstly characterized and identified, with the gene number of MGG_14621.6. Herein, sequence analysis showed that MoSDT1 contained 279-bp open reading frame (ORF) and encoded a 9.9 kDa protein, with a 16-amino acid signal peptide at the N-terminus. In addition, a predicted LxAR motif followed a signal peptide (SP), and a ZnF-C_2_H_2_ domain was located from amino acid 47 to amino acid 71 of MoSDT1 (Figure 1).

### 2.2. Effect of MoSDT1 Overexpression on Agronomic Traits in Rice

**Figure 2:**
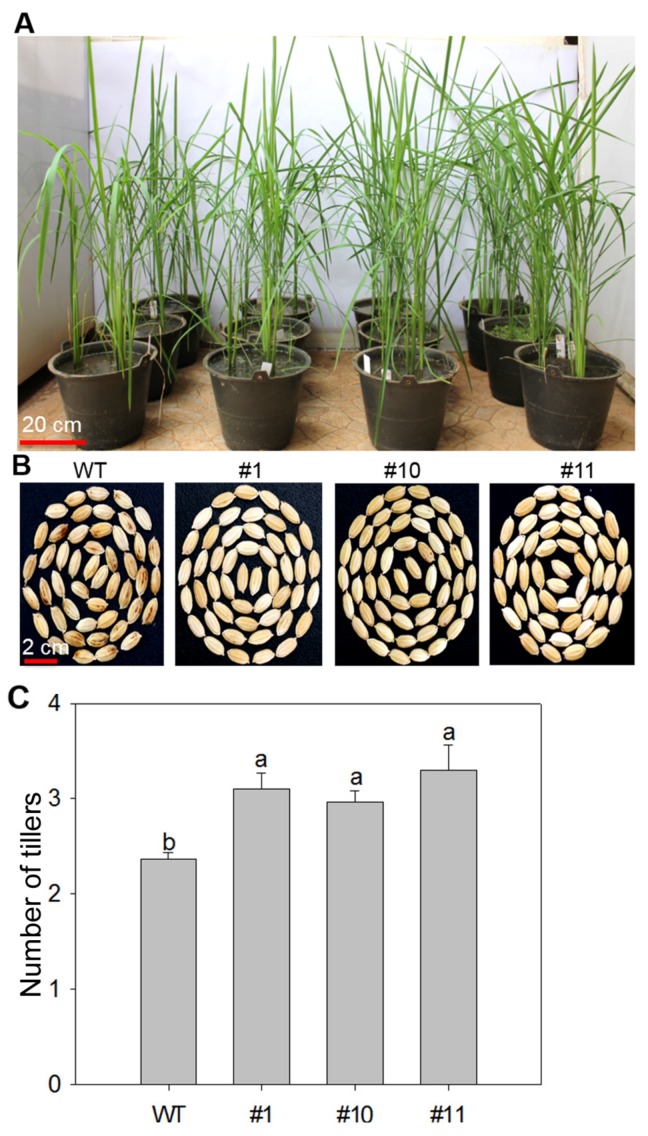
Agronomic traits of wild-type (WT) and three MoSDT1 overexpressing lines. (**A**) The picture showing the growth of WT and three MoSDT1 transgenic lines. Bar = 20 cm. (**B**,**C**) Kernel morphology (**B**) and the number of tillers (**C**) of WT and three MoSDT1 transgenic lines. Bar = 1 cm. Different letters represent statistically significant differences (*p* < 0.05).

In order to investigate the effect of MoSDT1 overexpression on agronomic traits in rice, WT and three MoSDT1 transgenic lines were grown under control conditions. The same as WT, three MoSDT1 overexpressing lines grew (Figure 2A), and the grains of three MoSDT1 transgenic lines were plump and smooth and spotless (Figure 2B). Notably, their tillers were significantly higher than those of WT (Figure 2C).

### 2.3. MoSDT1 Promotes Callose Deposition in Cell Wall of Leaf Sheath

**Figure 3:**
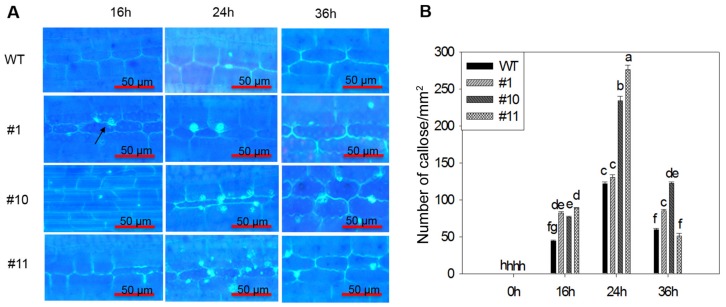
Callose deposition in sheath cells. (**A**) The pictures showing deposited callose in sheath cells. The bright spots marked with black arrows were deposited callose. (**B**) The number of callose deposition in sheath cells of WT and MoSDT1 transgenic lines. Different letters represent statistically significant differences (*p* < 0.05). Bars = 50 µm.

In order to reveal the role of MoSDT1 on the basic defense response in rice, WT and three MoSDT1 transgenic lines were infected by blast fungus strain 95234I-1b. The results of aniline blue staining showed that callose deposition was observed at 16 h after inoculation, increased at 24 h and began to decrease at 36 h in three MoSDT1 transgenic lines and WT sheath cells (Figure 3A,B). The average number of callose deposition in three MoSDT1 transgenic lines was significantly more than that in WT after 95234I-1b inoculation, indicating that MoSDT1 could promote callose deposition in cell wall.

### 2.4. MoSDT1 Regulates Cell Death and ROS Accumulation

**Figure 4:**
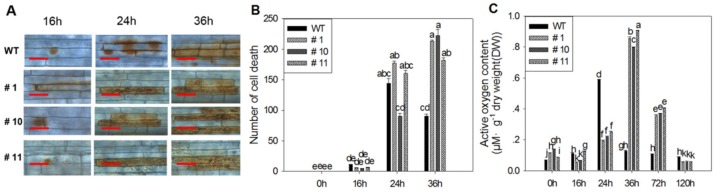
Cell death and ROS accumulation in plant leaves during rice–blast pathogen interaction. (**A**) DAB staining in leaf sheath cells. Bars = 25 µm. (**B**,**C**) The number of dead cells (**B**) and active oxygen content (**C**) in plant leaves during rice-blast pathogen interaction. Different letters represent statistically significant differences (*p* < 0.05).

Diaminobenzidine (DAB) staining showed that only browning appressorium cells were observed at 16 h after inoculation in the leaf sheaths of three MoSDT1 transgenic lines, while both browning appressorium cells and penetrating dead cells were found in infected WT leaf sheaths (Figure 4A,B). At 24 and 36 h after inoculation, the number of dead cells in leaf sheaths of three MoSDT1 transgenic lines increased with full of mycelia, significantly more than that in WT leaf sheaths (Figure 4A,B). This result indicated that MoSDT1 could induce the death of sheath cells in MoSDT1 transgenic lines, restrict the invasive mycelia to dead cells and prevent its expansion to adjacent cells.

Before pathogen inoculation, ROS accumulation in three MoSDT1 transgenic lines was significantly higher than that in WT (Figure 4A,B). After pathogen inoculation, ROS accumulation in leaves increased with time, reaching its peak at 36 h (Figure 4). Although ROS accumulation in three MoSDT1 transgenic lines was significantly lower at 16 and 24 h, but was significantly higher at 36 and 72 h than that in WT (Figure 4). This result was consistent with ROS accumulation pattern before and after BNS in hemibiotrophic fungal pathogens [7].

### 2.5. Overexpression of MoSDT1 Confers Improved Rice Blast Resistance

**Figure 5:**
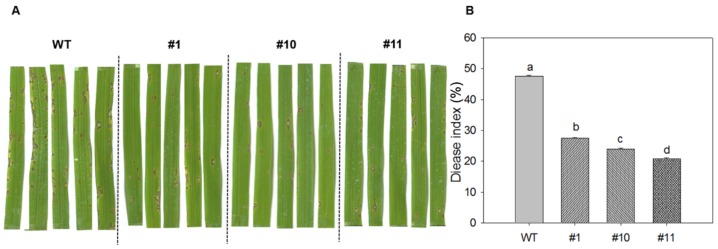
MoSDT1 overexpression confers improved rice blast resistance. (**A**) The symptom of rice blast resistance after 95234I-1b inoculation. (**B**) Disease index of rice blast resistance after 95234I-1b inoculation. Different letters represent statistically significant differences (*p* < 0.05).

After 95234I-1b inoculation, the symptom of local lesions was shown in Figure 5A. Generally, three MoSDT1 transgenic lines showed significant less lesions in the same area of leaves than WT. Consistently, the disease index of three MoSDT1 transgenic lines was significantly lower than that of WT (Figure 5B), indicating that overexpression of MoSDT1 conferred improved rice blast resistance.

### 2.6. Overexpression of MoSDT1 Regulates the Expression of Pathogenesis-Related Genes of Magnaporthe oryzae

**Figure 6:**
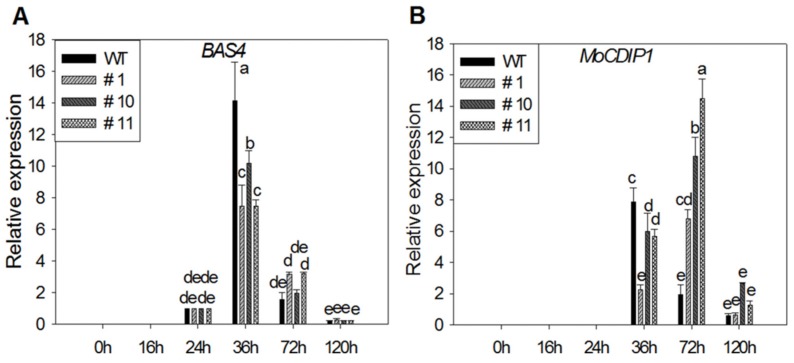
Expression of pathogenesis-related genes of *Magnaporthe oryzae* in WT and three MoSDT1 transgenic lines. (**A**,**B**) Relative expression levels of *MoBAS4* and *MoCDIP1*. Different letters represent statistically significant differences (*p* < 0.05).

Quantitative real time-PCR (qRT-PCR) analysis showed that the expression of MoBAS*4* in three MoSDT1 transgenic lines and WT reached its peak at 36 h after inoculation, with lower expression in three MoSDT1 transgenic lines than WT, and then began to decline (Figure 6A). The expression of *MoCDIP1* in three MoSDT1 transgenic lines and WT began to increase at 36 h with slightly lower expression in three MoSDT1 transgenic lines, peaked at 72 h with significantly higher expression in three MoSDT1 transgenic lines, and then began to decrease (Figure 6B).

### 2.7. Overexpression of MoSDT1 Modulates the Expression of Defense-Related Genes in Rice

**Figure 7:**
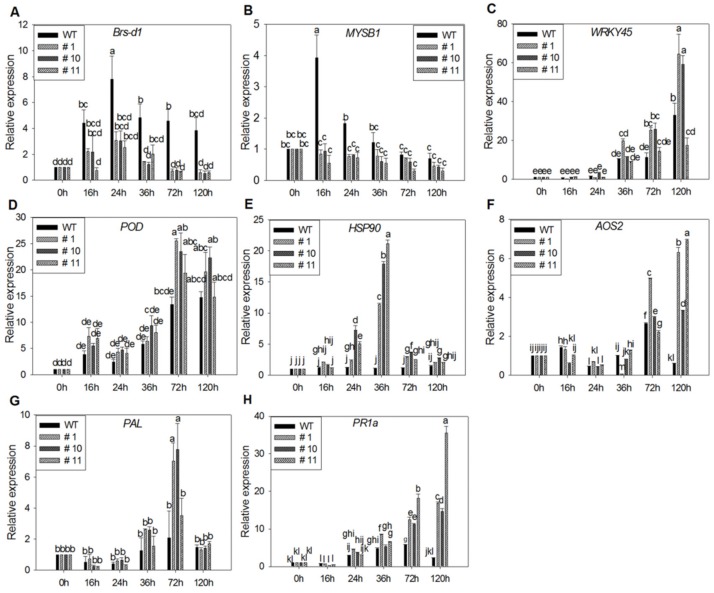
Expression of defense-related genes in MoSDT1 transgenic lines after 95234I-1binoculation. (**A**)-(**H**) Relative expression levels of *Bsr-d1* (**A**), *MYBS1* (**B**), *WRKY45* (**C**), *peroxidase* (*POD*) (**D**), *heat shock protein 90 (HSP90*) (**E**), *allenoxide synthase 2* (*AOS2*) (**F**), *phenylalanine ammonia lyase* (*PAL*) (**G**) and *pathogenesis-related protein 1a* (*PR1a*) (**H**) between WT and three MoSDT1 transgenic lines. Different letters represent statistically significant differences (*p* < 0.05).

After 95234I-1b inoculation, the expression of *Bsr-d1 and MYBS1* increased from 16 to 24 h and decreased from 24 to 120 h, with significant lower expression in three MoSDT1 transgenic lines than that in WT (Figure 7A,B). Generally, the expression of *WRKY45*, *peroxidase* (*POD*), *heat shock protein 90* (*HSP90*), *allenoxide synthase 2* (*AOS2*), *phenylalanine ammonia lyase* (*PAL*), *pathogenesis-related protein 1a* (*PR1a*) were higher after inoculation, peaking at 36 to 120 h, with significant higher expression level in three MoSDT1 transgenic lines than that in WT (Figure 7C–H). These results indicated that MoSDT1 overexpression had significant effect on the expression of defense-related genes in rice.

### 2.8. MoSDT1 Directly Regulates Bsr-d1 in Rice

**Figure 8:**
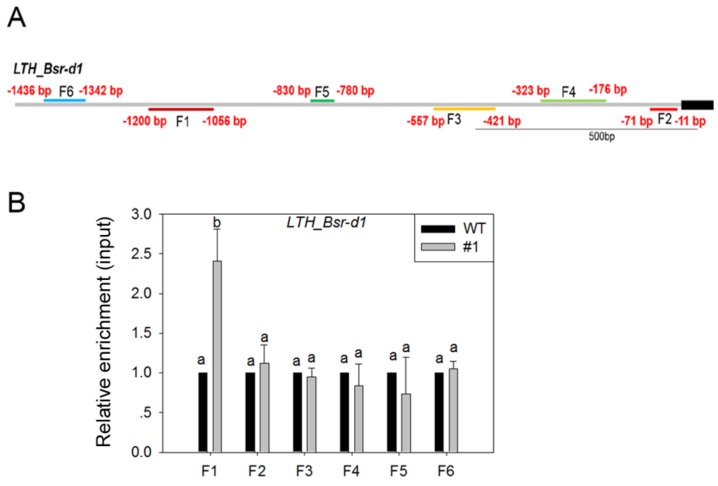
Relative enrichment of MoSDT1-GFP in the promoter of *OsBsr-d1*. (**A**) Chromatin immunoprecipitation-quantitative PCR (ChIP-qPCR) primer pairs in the promoter of *OsBsr-d1*. (**B**) Relative enrichment of MoSDT1-GFP in the promoter of *OsBsr-d1*. Different letters represent statistically significant differences (*p* < 0.05).

In order to clarify whether MoSDT1 directly regulates *Bsr-d1*, Chromatin immunoprecipitation-quantitative PCR (ChIP-qPCR) was performed. The results of ChIP-qPCR showed that there were significant differences in GFP enrichment level in the F1 region (−1200 bp to −1056 bp) in the promoter of *Bsr-d1* between MoSDT1 transgenic line #11 and WT, while no significant difference in the enrichment of other five regions (−1436 bp to −1342 bp, −830 bp to −780 bp, −557 bp to −421 bp, −323 bp to −176 bp, and −72 bp to −11 bp) in the promoter of *Bsr-d1* was observed (Figure 8A,B). This result indicated that MoSDT1 might directly bind to the promoter region of *Bsr-d1* to regulate its expression.

### 2.9. Overexpression of MoSDT1 Affects the Accumulation of Primary Metabolites in Rice

Based on the above results of defense response, the effect of MoSDT1 overexpression on primary metabolites in rice was further analyzed. Compared with WT, 6 differentially expressed primary metabolites in MoSDT1 were identified, including vanillic acid, L-saccharopine and L-tryptophan. L-tryptophan, L-tyrosine, usnic acid and 1-aminocyclopropane carboxylic acid (Table 1). The concentration of these metabolites was significantly higher in MoSDT1 transgenic line (Table 1).

## 3. Discussion

During plant–pathogen interaction, a pathogen secretes effector proteins to promote virulence and infection of the pathogen. Meanwhile, the plant recognizes these effector proteins to trigger plant defense response [6,11]. So far, effector proteins could be used as important genetic resources for transgenic improvement of plant disease resistance, including overexpression of PsCRN115 [4], CtNUDIX [7], MoSM1 [17] and so on.

Callose deposition is one of the direct defense responses of host plant to block invasion and expansion of pathogens [8,26,27]. Generally, weak and short ROS bursts occur during compatible plant–pathogen interaction, resulting in plant susceptibility; while strong and sustained ROS bursts occur in incompatible plant–pathogen interaction, triggering hypersensitive response (HR) to improve plant disease resistance [26,27,28]. ROS accumulation not only lead to oxidative damage to cell structures, but also plays a decisive role in BNS in many hemibiotrophic fungal pathogens [7]. This study showed that overexpression of MoSDT1 could activate callose deposition, ROS burst at 36–72 h and ROS scavenging at 120 h, and cell death after *Magnaporthe oryzae* inoculation. All these modulations might contribute to improve rice blast resistance of MoSDT1 transgenic lines in rice. Consistently, similar results have been reported on tobacco anthracnose disease against *Colletotrichum truncatum* [5], tobacco phytophthora blight against *Phytophthora sojae* [4], wheat stripe rust against *Puccinia striiformis* [17], and rice bacterial leaf blight against *Xanthomonas oryzae* [18]. In addition, overexpression of MoSDT1 had no significant negative effect on agronomic traits while improving disease resistance, indicating that pathogen effector proteins such as MoSDT1 could be used as important genetic resources for transgenic improvement of plant disease resistance.

BNS of *Magnaporthe oryzae* is the key period of disease occurrence [5,7,29]. *Macrophomina phaseolina* BAS3/NIP can be used to detect the BNS phase of the fungus [30]. Due to the crucial of MoBAS4 and MoCDIP in rice-pathogen interaction [6,21,22], the expression of *MoBAS4* and *MoCDIP1* wasanalyzed in MoSDT1 transgenic lines during rice–*Magnaporthe oryzae* interaction in this study. In addition, the peak of the expression of *MoBAS4* (36 h) was earlier than the peak of the expression of *MoCDIP1*(72 h), and the expression of *MoCDIP1* was even undetectable in MoSDT1 transgenic lines after 95234I-1b inoculation, indicating that 36 to 72 h might be BNS phase during rice–*Magnaporthe oryzae* interaction. Based on previous study that BNS could last for 36 h when *Magnaporthe oryzae* infects resistant varieties [30], overexpression of MoSDT1 in rice might delay BNS phase of *Magnaporthe oryzae*.

Based on genome-wide association study (GWAS) of 66 non-broad-spectrum resistant rice varieties, Li et al. [31] found that the natural variation in the promoter of *Bsr-d1* gene confers broad-spectrum blast resistance. *Bsr-d1* gene encodes a C_2_H_2_-type transcription factor, which promotes *POD* expression, regulates ROS accumulation, and is negatively regulated by a MYB family transcription factor MYBS1 [31]. Notably, the transcription factor MoSDT1 could combine to the promoter of *OsBsr-d1* gene and regulated its expression. In addition, the differential expression of *Bsr-d1* and *MYBS1* between MoSDT1 transgenic lines and WT was consistent with ROS accumulation before and after BNS [7]. We have to note that the binding site of MoSDT1 in the promoter of *OsBsr-d1* gene needs to be further clarified. The expression of *WRKY* genes are induced by *Magnaporthe oryzae* [32], SA and JA [33]. Overexpression of *OsWRKY45* improves disease resistance by regulating SA signaling pathway in rice [34,35]. POD, HSP90, JA biosynthesis-related AOS2, SA synthesis-related PAL, PR1a, are directly or indirectly regulated by WRKY transcription factors, thereby regulating plant stress response and growth [36,37,38,39]. *Ideal Plant Architecture 1* (*IPA1*) is a key gene regulating the formation of ideal plant architecture in rice. On the one hand, overexpression of IPA1 has ideal traits such as strong stalk, bigger panicle and higher yield. One the other hand, overexpression of *IPA1* can also improve rice blast resistance. The phosphorylation of IPA1 is the key hub between yield and resistance [39]. Under control conditions, IPA1 binds to promoters of panicle development-related genes such as *dense and erect panicle 1* (*DEP1*) to activate their expression, which is responsible for the establishment of ideal plant architecture and rice yield. Under *Magnaporthe oryzae* infection, the induced and phosphorylated IPA1 is more likely to bind to promoters of disease resistance-related genes such as *WRKY45*, promotes its expression, enhances immune response and improves disease resistance [39]. In this study, after 95234I-1b inoculation, the higher expression of the *WRKY45*, *POD*, *HSP90*, *AOS2*, *PAL* and *PR1a* genes in the MoSDT1 transgenic lines might be related to improved disease resistance. These results suggested MoSDT1 could directly or indirectly regulate the expression of these defense-related genes, resulting in the activation of multiple defense-related signaling pathways.

In this study, tyrosine and tryptophan were significantly increased in MoSDT1 transgenic line. They are known as aromatic amino acids, which are related to the synthesis of several secondary metabolites [23,24]. Among them, tyrosine is involved in the synthesis of plant pigments, and tryptophan is involved in the synthesis of plant hormones [40]. Tyrosine is the precursor of many special secondary metabolites (flavonoids, isoflavonoids, proanthocyanidins), which act as electronic carriers, antioxidants, attractants and defense metabolites with different physiological effects [40]. Therefore, overexpression of MoSDT1 increased accumulation of defense-related amino acids in rice, which might be related to the modulation of disease resistance. The accumulation of vanillic acid and L-saccharopine are closely related to the defense response of grapes against *Botrytis cinerea* [13]. Usnic acid has strong antimicrobial activity and have been found in many lichen species [41], so it has attracted wide attention in the pharmaceutical industry. The concentration of vanillic acid, L-saccharopine and usnic acid was higher in MoSDT1 transgenic line, indicating that these differentially expressed metabolites might be related to the improved defense response of MoSDT1 transgenic line. 1-Aminocyclopropane carboxylic acid can be converted to ET under the action of a specific enzyme [42]. The higher accumulation of 1-Aminocyclopropane carboxylic acid in MoSDT1 transgenic line might contribute to the enhanced defense response through modulation of ET. In addition, the variable importance in the projection (VIP) value in the orthogonal partial least-squares discriminant analysis (OPLS-DA) model was calculated to analyze the contribution of these metabolites to the classification. Notably, the VIP value of metabolites was more than 1, indicating their significant contribution to the classification. Based on the above analysis, we could conclude that the increased accumulation of these primary metabolites might be related to the improved disease resistance in MoSDT1 overexpressing lines. However, the in vivo roles of these metabolites in disease resistance against *Magnaporthe oryzae* needs to be further investigated.

Although the underlying mechanism of MoSDT1 in plant disease resistance was partially revealed, many questions remain unclear, which need to be further investigated in the future. Firstly, what are the other direct targets of MoSDT1 in rice besides *OsBsr-d1*? What motifs are responsible for the direct binding of MoSDT1 to the promoters? Secondly, how MoSDT1 regulates callose deposition, ROS accumulation and cell death in rice? In addition, are these differential primary metabolites directly involved in rice blast resistance in rice? Is ET directly or indirectly involved in MoSDT1-mediated rice blast resistance in rice? The further study of these questions will provide new information of MoSDT1-mediated rice blast resistance in rice.

Taken together, overexpression of MoSDT1 confers improved rice blast resistance in rice, through modulation of callose deposition, ROS accumulation, the expression of defense-related genes, and the accumulation of some primary metabolites.

## 4. Materials and Methods

### 4.1. Plant Materials and Growth

WT variety Ilimi, three MoSDT1 transgenic overexpressing lines (#1, #10 and #11) and rice blast strain 95,234 I-1b have been described previously [25]. Briefly, the 279 bp coding region of *MoSDT1* gene was amplified by PCR from rice blast strain 95234I-1b, using the primers with restriction sites (F: 5’-GTCGACTGGACGAGTACGAACAGCAC-3´, R: 5´-GGATCCATTGAGGTGAGGGTTTGGGG-3´). Then the PCR fragment was further cloned into the binary expression vector pCAMBIA1305.2 with eGFP and hygromycin resistance gene, producing *MoSDT1: eGFP*. After the overexpressed clone was confirmed in proper frame by sequencing, the *pMoSDT1::eGFP* plasmid was transformed into *Agrobacterium tumefaciens* EHA105 and further transformed to rice (Ilimi) callus [18]. More than 10 independent transgenic lines were obtained, identified and confirmed by PCR with the specific primers of hygromycin resistance gene, *eGFP* and *MoSDT1*. In addition, T2 and T3 generation lines were used for disease resistance analysis and similar results were obtained. Therefore, three independent transgenic lines (#1, #10 and #11) were used for further studies. All materials were obtained and preserved in our lab.

After 20 min of sterilization with 75% absolute ethanol and 2% hypochlorite respectively, the seeds of *MoSDT1* transgenic lines (#1, #10 and #11) and WT were germinated in 5% hygromycin aqueous solution and steric water, respectively, according to Liu et al. [43] described previously. Then the seeds were sown in paddy soil and humus with the ratio of 3:1 in plastic buckets and plates, each treatment was repeated three times. The seedlings in plastic barrels were cultivated to maturity according to conventional management in the greenhouse, which was controlled at 28 °C/26 °C (day/night) with 16h light (about 250 mmol·m^−2^·s^−1^)/8 h dark and a relative humidity of 50%. The agronomic traits of 10 individual plants were randomly investigated for each treatment.

In this study, large size of randomization was applied for the experiment. All experiments were performed with at least three biological replicates. In addition, every biological replicate was constituted of one group of leaves, and whole leaves of 10 individual plants in the same leaf part and the same sizes were randomly collected for analysis.

### 4.2. Chemical Reagents

Reagents for metabonomics analysis, including ammonium acetate (NH_4_AC), ammonium hydroxide (NH_4_OH), ammonium fluoride (NH_4_F), and formic acid (FA) were purchased from Sigma Aldrich (St Louis, MO, USA). Acetonitrile was purchased from Merck (KGaA, Darmstadt, Germany).

### 4.3. Analysis of MoSDT1 Sequence

Bioinformatic analysis of *MoSDT1* sequence was performed using ExPASY proteomics server (http://www.expasy.ch/tools/pi_tool.html), MultiLoc/TargetLoc (http://www-bs.informatik.uni-tuebingen.de/Services/MultiLoc) and InterProScan (http://www.ebi.ac.uk/Tools/InterProScan/). Multiple alignment software T-Coffee (http://tcoffee.crg.cat/apps/tcoffee/play?name=regular) and GeneDoc were also used for analysis [44].

### 4.4. Inoculation of Magnaporthe Oryzae

Pathogen inoculation was performed as Li et al. [31] described. *Magnaporthe oryzae* strain 95234I-1b was cultured in Potato Dextrose Agar (PDA) medium plate at 28 °C until the hyphae grew into a culture dish. The hyphae was broken and transferred to PDA liquid medium at 28 °C and 160 r/min for 5–7 days. Then the liquid pathogen was evenly applied to the prune juice medium (40 mL prune juice, 5 g lactose, 1 g yeast powder, 15 g agar powder, 1000 mL sterile water), and cultured under light at 25 °C light for 7–10 days. The spore suspension was diluted to 1 × 10^5^/mL with sterile water and sprayed on rice leaves at three-leaf-stage. The leaves were moisturized at 28 °C for 24 h and then moved to greenhouse for growth. Foot leaves were taken at 0, 16, 24, 36, 72, 120 and 14 days, and stored at −80 °C. On the 7th day after inoculation, 120 plants were randomly investigated for disease severity and disease index.

### 4.5. Observation of Callose Staining

Callose staining was performed as Zhang et al. [4] described. Briefly, 6 fresh leaf sheaths at 0, 16, 24 and 36 h after of *Magnaporthe oryzae* 95234I-1b inoculation, respectively. After cleaning with 95% ethanol, plant leaves were incubated in ethanol-emulsifiable solution (phenol: glycerol: lactic acid: water: ethanol = 1:1:1:1:8) at 65°C until the green color was completely removed, washed with 50% ethanol and sterilized water. After staining in 0.1% aniline blue for 1 h, callose was observed and photographed under DMI4000B fluorescence inverted microscope (Leica Camera AG, Wetzlar, Germany), and the number of callose was counted in each treatment according to 20 field of vision.

### 4.6. Determination of ROS Accumulation

ROS accumulation was assayed as Perez and Rubio [45] described. Briefly, 100 mg plant fresh leaves were grinded to powder with liquid nitrogen and mixed with 500 μL PBS buffer solution. After centrifugation at room temperature for 15 min, the supernatant was used for ROS assay with ROS Enzyme Linked Immunosorbent Assay (ELISA) Kit (Jiangsu Enzyme Immunity Industry Co., Ltd., Yancheng, China). The value of OD_450 nm_ was determined in Varioskan LUX-type multifunctional enzyme instrument (ThermoFisher, Waltham, MA, USA), ROS content of the samples was calculated according to the standard curve. The experiment was repeated three times.

### 4.7. Observation and Statistical Analysis of Dead Cells

Dead cells were analyzed as Thordal-Christensen et al. [46] described. Briefly, 10 fresh leaf sheaths of each sample were rinsed twice with sterile water and stained in 1 mg/mL DAB solution (pH 5.8) for 24 h, the samples were incubated in 100% ethanol until the green color was completely removed. A total of 20 visual fields were observed under DMI4000B fluorescence inverted microscope (Leica, Germany), and the number of dead cells was counted according to the photos. The experiment was repeated three times.

### 4.8. Analysis of Expression of Pathogenic Genes of Magnaporthe oryzae and Plant Defense-Related Genes

Briefly, 100 mg plant fresh leaves were ground to powder with liquid nitrogen, and then total RNA was extracted using RNA Extraction Kit (RNAiso Plus Kit, TransGen Biotechnology Co., Ltd., Beijing, China). After the detection of the concentration and integrity, the total RNA was used for cDNA synthesis by TransScript^®^ All-in-one First-Strand cDNA Synthesis SuperMix for qPCR (TransGen Biotechnology Co., Ltd., Beijing, China). The qRT-PCR was then performed using 2×TransStart^®^ Top/Tip Green qPCR SuperMix (TransGen Biotechnology Co., Ltd., Beijing, China) in CFX96 Quantitative PCR Instrument (Bio-Rad, Hercules, CA, USA) to analyze the expression of pathogenic genes of *Magnaporthe oryzae* (*MoBAS4* and *MoCDIP5*) and plant defense-related genes (*OsBsr-d1*, *OsMYBS1*, *OsWRKY45*, *OsPOD*, *OsHSP90*, *OsAOS2*, *OsPAL* and *OsPR1*). The primers were designed using Primer 5 software (http://www.premierbiosoft.com/primerdesign/ or https://www.rar8.net/Soft/133451.html), and the specificity of these primers were confirmed by electrophoresis detection of PCR product and the amplification curve of qRT-PCR through CFX Manger™ software. Primer sequences are shown in Table S1. The CFX Manger™ software attached in CFX96 Quantitative PCR Instrument (Bio-Rad, USA) was used to analyze the relative gene expression according to 2^−ΔΔ*C*t^ method [47] (Table S2).

### 4.9. ChIP-qPCR

ChIP-qPCR was performed with MoSDT1 transgenic line (#11) and WT as materials as Li et al. [31] previously described. Six pairs of primers were designed to amplify every 300 bp fragment in the promoter sequences of genes *OsBsr-d1* and *OsMYBS1*, respectively. The relative expression levels were normalized to the promoter of the *Ubiquitin* gene (LOC_Os03g13170). No addition of antibody (NoAb) was served as the negative control. According to the formula: Fold enrichment = 2^−ΔΔ*C*t^, the enrichment of GFP protein in each region was obtained.

### 4.10. Ultra-Performance Liquid Chromatography (UHPLC)-Quadrupole (Q)-Time of Flight (TOF) Mass Spectrometry (MS)

Plant fresh leaves were grinded to powder with liquid nitrogen and mixed with 1 mL methanol:acetonitrile:aqueous solution (2:2:1, *v:v:v*). After twice of frozen-dried for 60 s and cryogenic ultrasound for 30 min, the mixture was placed at −20 °C for 1 h to precipitate protein. After filtration by the filter tube and centrifugation at 14,000 rcf, 4 °C for 20 min, the supernatant was frozen-dried and stored at −80 °C. The samples were then analyzed using an UHPLC (1290 Infinity LC, Agilent Technologies, Santa Clara, CA, USA) coupled to a Q-TOF (AB Sciex TripleTOF 6600) and a 2.1 mm × 100 mm ACQUIY UPLC BEH 1.7 µm column (Waters, Dublin, Ireland). Metabolites were identified by accuracy *m/z* value <25 ppm, and Metlin (https://metlin.scripps.edu) database of MS/MS spectra was used as the strandard. The VIP value in OPLS-DA model was calculated to analyze the contribution to the classification. Metabolites with the VIP value >1 was further applied to Student’s t-test, and *p* < 0.05 indicated statistically significant.

### 4.11. Statistical Analysis

All data were analyzed by IBM SPSS Modeler 17.0 software (http://www-01.ibm.com/software/analytics/spss/), and the data were expressed as mean ± SD. Statistical significance between WT and transgenic lines was determined by one-way analysis of variance (ANOVA) and adjusted by Bonferroni method. All the tests were two-sided, and Different letters represent statistically significant differences (*p* < 0.05). The figures were obtained by SigmaPlot 10.0 software (https://systatsoftware.com/).

## Figures and Tables

**Table 1:** Differentially expressed metabolites between WT and MoSDT1 transgenic line.

Metabolites	Fold Change (#11/WT)	*p*	VIP
Vanillic acid	1.40 ± 0.20	0.00005	2.36062
L-saccharopine	1.47 ± 0.23	0.00034	2.16403
L-tryptophan	2.21 ± 0.61	0.00176	6.30722
L-tyrosine	2.07 ± 0.53	0.00191	1.02201
Usnic acid	2.16 ± 0.58	0.00104	8.11674
1-aminocyclopropane carboxylic acid	1.21 ± 0.11	0.01173	2.43278

## Supplementary Materials

Supplementary Materials can be found at https://www.mdpi.com/1422-0067/20/19/4762/s1.
