# Supplementary material for: Overexpression of Magnaporthe Oryzae Systemic Defense Trigger 1 (MoSDT1) Confers Improved Rice Blast Resistance in Rice
Source: Int J Mol Sci. 2019 Sep 25;20(19):4762. doi: 10.3390/ijms20194762 (PMC6802482; doi:10.3390/ijms20194762)
Supplement: Supplementary file 1 [file ijms-20-04762-s001.pdf]

Supplementary materials:

Table S1. Primers used for qRT-PCR.

Gene	Primer	Sequence
OsHSP90	F	5′-CAAGTCGGACCTCGTCAACA-3′
	R	5′-TCTCAGCAACAAGGTAGGCG-3′
OsPR1a	F	5′-GCTACGTGTTTATGCATGTATGG-3′
	R	5′-TCGGATTTATTCTCACCAGCA-3′
OsPAL	F	5′-TCACAAGCTCAAGCACCATC-3′
	R	5′-CTCACCAAGCTTCTTGGCAT-3′
OsAOS2	F	5′-GGAGGAAGCTGCTGCAATAC-3′
	R	5′-GTGTCGTACCGGAGGAAGAG-3′
OsBsr-d1	F	5′-CAAGCGCTGCTCGACCCGAC-3′
	R	5′-GGTACTCCTCCTTGTTGATCGCCG-3′
OsMYBS1	F	5′-GCAAAACCGAGGAGGTAGAGA-3′
	R	5′-AGTATTGGTTTTGCGGCTGG-3′
OsWRKY45	F	5′-CGGGTAAAACGATCGAAAGA-3′
	R	5′-TTTCGAAAGCGGAAGAACAG-3′
OsPOD	F	5′-TCGCCTGATGCAAATCGGTA-3′
	R	5′-TTGATGCACAACCAACCAACC-3′
OsActin	F	5′-GAGTATGATGAGTCGGGTCCAG-3′
	R	5′-ACACCAACAATCCCAAACAGAG-3′
OsActin	F	5′-GTCCTCTTCCAGCCTTCCTT-3′
	R	5′-TACCACCACTGAGAACGATGT-3′
MoBAS4	F	5′-CGACAACAACAGTGCAGAGAA-3′
	R	5′-TCTCCCAGATAGTGCAGTCGT-3′
MoCDIP1	F	5′-GTAACCGGAATGGTCGTGGT-3′
	R	5′-ATGCGCACGTTGAAGTTGTC-3′
MoTubulin	F	5′-ACCACATTGGAGCACTCTGAC-3′
	R	5′-GTGATTGAGGAAACGACCTGA-3′
MoEF1α	F	5′-CAAGCTGAAGGGTATTGACCA-3′
	R	5′-TTGTCAAGAACCCAGGCATAC-3′
OsBsr-d1	F1F	5′-GGTGTATAGTGGTGGTGTATTGC-3′
	F1R	5′-TTTGTTAGGACAGAGGAATTACGC-3′
	F2F	5′-GCTCGCTGCTGCTGCTAC-3′
	F2R	5′-GTTGCCGTCGTCGTAAAACC-3′
	F3F	5′-GCAAACAGAAAGTACAGGTTCATCC-3′
	F3R	5′-GGGCGTGGTTTTGGTGTGG-3′
	F4F	5′-ACTAAAAGCCAGCCCACACAG-3′
	F4R	5′-AGGGATGGAGAGAAATCAAACCG-3′
	F5F	5′-GTCTAGCATCCACCGTTCCAC-3′
	F5R	5′-CGAATAAGCGAAACGATGAGTCC-3′
	F6F	5′-CGCCTTTCTACACGCAACG-3′
	F6R	5′-AGCACCCTACTGAAGTGATGAAC-3′
OsUBQ	ChIPF	5′-TCGGAGACCGTGCTAGGTTT-3′
	ChIPR	5′-GCCAGCGCCCATCGATT-3′

Table S2. The raw data of qRT-PCR.

		Ct		
OsActin	**Line**	**Rep1**	**Rep2**	**Rep3**
0 h	WT	23.03	23.65	23.39
	1#	22.87	22.94	22.81
	10#	23.78	23.66	24.00
	11#	23.98	24.21	24.24
16 h	WT	24.04	24.11	24.25
	1#	25.32	25.63	25.78
	10#	23.28	23.43	23.87
	11#	22.66	23.43	23.13
24 h	WT	22.79	22.75	22.67
	1#	22.42	22.31	22.21
	10#	22.45	22.33	22.54
	11#	22.62	23.00	22.32
36 h	WT	22.53	22.75	22.38
	1#	22.49	22.58	22.37
	10#	22.56	22.19	22.57
	11#	22.68	22.58	22.81
72 h	WT	21.94	21.75	21.77
	1#	21.47	22.08	22.48
	10#	22.21	21.57	22.24
	11#	21.79	22.02	21.98
120 h	WT	21.90	22.13	21.82
	1#	22.17	22.35	22.47
	10#	22.24	22.33	22.09
	11#	22.57	22.67	22.78
OsHSP90	**Line**	**Rep1**	**Rep2**	**Rep3**
0 h	WT	24.06	24.78	24.22
	1#	22.88	23.08	22.24
	10#	24.14	24.09	24.60
	11#	24.14	24.25	24.14
16 h	WT	24.95	24.72	24.94
	1#	24.23	24.69	24.10
	10#	23.17	23.00	23.75
	11#	22.58	23.19	22.84
24 h	WT	23.59	23.46	23.35
	1#	21.27	21.09	20.52
	10#	20.07	20.10	20.04
	11#	20.44	20.56	20.03
36 h	WT	23.40	23.65	23.02
	1#	18.97	19.19	18.33
	10#	18.78	18.52	18.95
	11#	18.37	18.20	18.39
72 h	WT	22.68	22.80	22.39
	1#	19.98	20.63	20.35
	10#	20.74	20.14	20.93
	11#	20.63	20.70	20.56
120 h	WT	22.47	22.52	22.18
	1#	21.15	21.46	20.84
	10#	21.20	21.25	21.22

	11#	21.70	21.74	21.58
OsPR1a	**Line**	**Rep1**	**Rep2**	**Rep3**
0 h	WT	31.57	32.46	32.03
	1#	30.28	30.50	29.93
	10#	32.72	32.13	32.76
	11#	30.46	30.32	29.72
16 h	WT	33.05	33.18	33.19
	1#	33.32	33.73	33.41
	10#	33.98	33.71	34.92
	11#	30.34	30.72	29.69
24 h	WT	29.80	29.96	29.69
	1#	27.65	27.71	27.07
	10#	29.43	29.01	29.46
	11#	27.46	27.45	26.14
36 h	WT	28.71	29.40	28.76
	1#	26.86	27.06	26.35
	10#	28.85	28.40	29.04
	11#	26.48	26.00	25.55
72 h	WT	27.92	27.99	27.89
	1#	25.36	25.87	25.96
	10#	27.62	26.59	27.50
	11#	23.95	24.10	23.27
120 h	WT	29.25	29.72	29.17
	1#	25.52	25.78	25.57
	10#	27.34	27.03	26.84
	11#	23.81	23.57	23.26
OsPAL	**Line**	**Rep1**	**Rep2**	**Rep3**
0 h	WT	22.83	23.42	23.03
	1#	23.93	23.72	23.60
	10#	23.38	23.43	23.55
	11#	22.84	22.74	22.96
16 h	WT	26.52	26.50	26.71
	1#	26.06	26.76	26.32
	10#	25.14	25.09	24.86
	11#	24.62	24.84	24.29
24 h	WT	24.39	24.38	24.06
	1#	24.16	24.26	23.97
	10#	22.27	22.86	22.17
	11#	22.87	23.59	23.17
36 h	WT	23.56	23.63	23.26
	1#	22.03	22.05	21.76
	10#	20.73	20.52	20.59
	11#	20.41	20.17	20.25
72 h	WT	23.30	23.02	22.76
	1#	20.07	20.32	20.71
	10#	18.58	18.09	18.56
	11#	18.50	18.33	18.50
120 h	WT	21.31	21.30	20.68
	1#	22.95	22.48	22.88
	10#	21.76	21.69	21.38
	11#	20.65	20.65	20.54

OsAOS2	Line	Rep1	Rep2	Rep3
0 h	WT	24.07	25.15	24.55
	1#	25.35	25.55	25.18
	10#	25.06	25.13	25.30
	11#	24.85	25.12	25.00
16 h	WT	24.68	24.99	24.86
	1#	27.72	27.65	27.63
	10#	25.29	25.87	25.72
	11#	23.64	24.20	23.91
24 h	WT	24.93	25.40	24.95
	1#	25.51	25.52	25.05
	10#	24.98	24.89	25.17
	11#	24.49	24.79	24.02
36 h	WT	23.54	24.26	23.51
	1#	28.60	29.00	28.75
	10#	24.17	23.92	24.11
	11#	23.15	23.14	23.23
72 h	WT	21.55	21.82	21.57
	1#	21.62	22.38	22.53
	10#	21.96	21.42	21.98
	11#	21.59	21.69	21.59
120 h	WT	23.56	24.52	23.70
	1#	21.89	22.39	22.20
	10#	21.77	22.08	21.66
	11#	20.65	20.81	20.72
OsActin	**Line**	**Rep1**	**Rep2**	**Rep3**
0 h	WT	23.81	23.66	23.47
	1#	23.02	23.85	23.79
	10#	24.92	24.62	25.06
	11#	24.41	25.26	24.83
16 h	WT	25.73	25.70	25.53
	1#	24.59	24.64	24.69
	10#	24.92	24.03	24.75
	11#	24.53	24.37	24.29
24 h	WT	23.43	23.41	23.73
	1#	22.50	22.89	22.62
	10#	23.09	23.38	23.19
	11#	23.18	23.13	23.25
36 h	WT	23.09	23.02	23.05
	1#	23.08	22.91	23.12
	10#	23.65	23.51	23.59
	11#	23.72	24.03	24.09
72 h	WT	21.25	21.41	21.42
	1#	21.18	21.32	21.63
	10#	21.69	21.86	21.38
	11#	21.04	21.26	21.13
120 h	WT	21.46	21.48	21.40
	1#	21.13	21.03	20.96
	10#	21.43	21.27	21.14
	11#	21.32	21.33	21.26
OsBsr-dl	**Line**	**Rep1**	**Rep2**	**Rep3**

0 h	WT	32.66	31.53	32.33
	1#	32.32	32.43	31.50
	10#	29.56	34.47	32.12
	11#	32.35	32.46	31.38
16 h	WT	32.05	32.28	32.05
	1#	32.48	32.28	31.38
	10#	31.06	31.71	31.10
	11#	32.87	31.38	32.20
24 h	WT	29.14	29.15	29.23
	1#	30.05	29.43	29.51
	10#	27.08	31.26	28.38
	11#	29.32	29.13	28.92
36 h	WT	29.49	29.42	29.27
	1#	31.84	31.03	30.29
	10#	28.44	32.98	30.14
	11#	30.27	29.74	31.41
72 h	WT	27.85	27.69	27.69
	1#	30.68	30.03	30.65
	10#	27.42	31.58	28.81
	11#	29.51	29.03	28.40
120 h	WT	28.34	28.30	27.80
	1#	30.44	30.72	30.18
	10#	28.41	31.39	29.08
	11#	29.48	29.47	29.08
OsMYBS1	**Line**	**Rep1**	**Rep2**	**Rep3**
0 h	WT	33.74	33.61	33.03
	1#	32.40	32.59	33.26
	10#	34.01	33.73	33.88
	11#	32.81	33.11	33.74
16 h	WT	33.35	33.53	33.76
	1#	34.20	34.11	34.08
	10#	33.82	32.89	34.63
	11#	34.31	34.33	33.14
24 h	WT	32.46	32.65	32.29
	1#	32.40	32.19	32.22
	10#	32.58	32.75	32.31
	11#	32.10	32.21	32.12
36 h	WT	33.00	32.09	32.97
	1#	32.20	32.56	33.39
	10#	33.03	33.12	34.11
	11#	34.03	32.65	33.31
72 h	WT	31.62	31.36	31.44
	1#	31.12	30.63	31.54
	10#	31.16	31.63	31.68
	11#	31.28	31.42	31.31
120 h	WT	31.34	32.31	31.93
	1#	31.09	31.65	31.66
	10#	31.44	31.62	31.58
	11#	32.01	31.33	31.24
OsWRKY45	**Line**	**Rep1**	**Rep2**	**Rep3**
0 h	WT	31.69	30.63	31.26

	1#	32.08	32.17	32.18
	10#	33.56	33.77	33.89
	11#	32.08	31.78	31.92
16 h	WT	33.51	33.97	34.44
	1#	35.35	34.64	35.14
	10#	34.02	33.13	33.42
	11#	31.59	31.28	31.19
24 h	WT	30.33	30.43	30.77
	1#	31.46	31.85	31.40
	10#	30.57	30.46	30.43
	11#	30.71	30.30	30.51
36 h	WT	27.54	26.72	27.42
	1#	27.70	27.07	27.18
	10#	28.79	29.11	28.85
	11#	28.15	27.44	28.06
72 h	WT	25.41	25.62	25.40
	1#	25.60	24.85	25.47
	10#	26.02	26.06	25.49
	11#	24.82	24.26	24.09
120 h	WT	23.88	23.97	24.18
	1#	23.79	23.58	23.58
	10#	24.32	24.33	24.19
	11#	24.41	24.43	24.20
OsPOD	**Line**	**Rep1**	**Rep2**	**Rep3**
0 h	WT	33.22	33.07	33.31
	1#	34.64	34.62	34.11
	10#	34.93	35.71	34.98
	11#	34.91	35.28	35.67
16 h	WT	35.74	35.71	35.97
	1#	35.93	34.23	35.16
	10#	34.67	33.38	34.47
	11#	34.00	33.25	33.55
24 h	WT	36.96	36.08	36.95
	1#	34.72	34.13	34.12
	10#	34.75	34.98	34.70
	11#	34.50	34.58	34.72
36 h	WT	33.20	33.09	33.19
	1#	35.96	35.23	35.29
	10#	35.01	35.23	35.13
	11#	38.60	38.17	38.34
72 h	WT	34.23	34.75	34.69
	1#	35.28	38.01	36.13
	10#	35.41	37.50	34.24
	11#	36.47	35.10	36.64
120 h	WT	35.10	34.68	35.38
	1#	37.51	35.70	33.14
	10#	33.80	35.83	36.40
	11#	33.23	36.35	38.64
MoEF1a	**Line**	**Rep1**	**Rep2**	**Rep3**
0 h	WT	28.45	28.23	27.73
	1#	28.62	28.34	28.24

	10#	29.08	29.34	28.37
	11#	29.56	29.43	29.65
16 h	WT	27.23	27.34	27.30
	1#	29.36	29.53	29.23
	10#	27.24	26.49	26.71
	11#	28.80	28.81	28.68
24 h	WT	26.20	26.06	26.35
	1#	27.68	27.66	27.29
	10#	26.11	26.02	26.03
	11#	27.24	27.08	27.35
36 h	WT	26.28	26.29	26.33
	1#	27.36	27.27	27.59
	10#	27.19	27.29	27.37
	11#	27.07	27.27	27.12
72 h	WT	27.45	27.51	27.33
	1#	27.16	26.96	27.11
	10#	29.73	29.75	29.68
	11#	23.98	24.20	24.08
120 h	WT	20.80	21.24	20.91
	1#	23.07	22.96	23.00
	10#	20.70	20.70	20.43
	11#	22.22	21.95	22.17
MoBAS4	**Line**	**Rep1**	**Rep2**	**Rep3**
0 h	WT	ND	ND	ND
	1#	ND	ND	ND
	10#	ND	ND	ND
	11#	ND	ND	ND
16 h	WT	ND	ND	ND
	1#	ND	ND	ND
	10#	ND	ND	ND
	11#	ND	ND	ND
24 h	WT	33.58	33.01	33.16
	1#	34.23	33.83	34.01
	10#	33.15	32.43	32.79
	11#	34.17	33.93	34.23
36 h	WT	29.42	29.74	29.54
	1#	31.01	31.05	31.02
	10#	30.80	30.60	30.65
	11#	31.27	31.13	31.04
72 h	WT	34.76	34.26	32.82
	1#	32.09	31.37	32.22
	10#	35.52	35.51	35.50
	11#	29.16	29.36	29.34
120 h	WT	30.11	30.25	30.32
	1#	31.38	31.28	31.06
	10#	29.68	29.39	29.45
	11#	31.07	31.01	31.09
MoTubulin	**Line**	**Rep1**	**Rep2**	**Rep3**
0 h	WT	28.75	28.44	28.60
	1#	27.29	27.33	27.42
	10#	26.98	27.12	27.06

	11#	26.41	26.21	26.30
16 h	WT	28.91	28.67	28.68
	1#	26.08	25.59	25.77
	10#	27.42	27.39	27.39
	11#	27.71	27.94	28.02
24 h	WT	25.07	25.55	25.19
	1#	25.72	25.53	25.74
	10#	26.11	26.00	26.11
	11#	27.36	27.30	27.71
36 h	WT	27.90	27.85	27.74
	1#	26.42	26.24	26.94
	10#	26.68	27.00	27.04
	11#	30.01	29.56	29.55
72 h	WT	24.15	24.09	23.73
	1#	26.08	26.35	26.27
	10#	26.15	26.13	26.19
	11#	28.05	28.06	28.08
120 h	WT	25.04	25.06	25.52
	1#	25.60	25.82	25.65
	10#	26.71	26.55	26.65
	11#	25.87	25.03	25.40
MoCDIP1	**Line**	**Rep1**	**Rep2**	**Rep3**
0 h	WT	ND	ND	ND
	1#	ND	ND	ND
	10#	ND	ND	ND
	11#	ND	ND	ND
16 h	WT	ND	ND	ND
	1#	ND	ND	ND
	10#	ND	ND	ND
	11#	ND	ND	ND
24 h	WT	36.63	36.33	36.72
	1#	34.26	34.65	34.86
	10#	37.23	37.53	37.68
	11#	34.76	34.67	34.76
36 h	WT	35.82	35.62	35.05
	1#	34.19	34.19	34.58
	10#	35.89	35.61	35.86
	11#	34.69	34.49	34.28
72 h	WT	34.03	33.21	34.21
	1#	32.11	32.51	32.61
	10#	34.20	34.14	34.12
	11#	31.51	31.86	31.12
120 h	WT	37.23	36.65	36.25
	1#	35.75	35.13	35.38
	10#	36.38	36.67	36.86
	11#	32.42	32.28	32.57

Note: ND, not determined.
